# Glasgow PrognosticScore as a Predictor of BevacizumabEfficacy in the First-line Treatment with Metastatic Colorectal Cancer

**DOI:** 10.7150/jca.31182

**Published:** 2019-11-15

**Authors:** Yuanyuan Huang, Weiyu Li, Qi Quan, Bei Zhang, Qiong Yang

**Affiliations:** 1 VIP Region, Sun Yat-sen University Cancer Center, Guangzhou, Guangdong, 510060, R.P. China; 2 State Key Laboratory of Oncology in South China, Sun Yat-sen University Cancer Center, Guangzhou, Guangdong, 510060, R.P. China; 3 Collaborative Innovation Center for Cancer Medicine, Guangzhou, Guangdong, 510060, R.P. China; 4 Department of Oncology, Sun Yat-Sen Memorial Hospital, Sun Yat-Sen University, Guangzhou 510120, R.P. China; 5 Guangdong Provincial Key Laboratory of Malignant Tumor Epigenetics and Gene Regulation, Sun Yat-Sen Memorial Hospital, Sun Yat-Sen University, Guangzhou 510120, China; 6 Medical Research Center, Sun Yat-Sen Memorial Hospital, Sun Yat-Sen University, Guangzhou 510120, China

**Keywords:** Glasgow prognostic score, bevacizumab, first-line therapy, metastatic colorectal cancer

## Abstract

**Background:** Inflammation might play an important role in promoting cancer growth partly by affecting tumor angiogenesis. We explored the role of Glasgow prognostic score (GPS) in metastatic colorectal cancer patients receiving first-linebevacizumab.

**Methods:** All consecutive metastatic colorectal cancer patients treated with first-line chemotherapy plus or not plus bevacizumab were eligible. Pre-treatment GPS were collected for all cases.

**Results:** In the chemotherapy group for patients with GPS of 0, 1 and 2, median progression-free survival (PFS) was 8.67, 8.10, and 8.27months, respectively (P = 0.17). Median overall survival (OS) was 24.87, 23.30, and 17.93months, respectively (P = 0.04). In the bevacizumab group, median PFS was 11.83, 8.10, and 6.87 months, respectively (P = 0.01), and median OS was 30.80, 19.47, and 18.67 months, respectively (P = 0.03).In whole group patients with a GPS of 0, both PFS and OS were in favor of patients treated with bevacizumab plus chemotherapy compared with who treated with chemotherapy alone (PFS 11.83 vs. 8.67 months, p=0.03; OS 30.80 vs. 24.87 months, p=0.04).

**Conclusion:** GPS of 0 was correlated with good prognosis. Bevacizumab added a survival advantage only in metastatic colorectal cancer patients with a GPS of 0.

## Introduction

In the worldwide, colorectal cancer is the third most commonly diagnosed cancer in males and the second most common in females, with an estimated 1.4 million cases and 693,900 deaths occurring in 2012 1. It is reported that metastatic disease accounted for 40%-50% of newly diagnosed colorectal cancer 2. Bevacizumab plus chemotherapy was shown to prolong survival of metastatic colorectal cancer (mCRC) patients, leading to approval for first line treatment in association with standard chemotherapy 3,4.

Until now, angiogenic biomarkers can't effectively predict who will benefit from bevacizumab treatment, though bevacizumab is a drug designed for its antiangiogenic effects.^5^ Recent studies have found that tumor infiltration inflammatory cells can induce angiogenesis mediated by inflammatory mediators and cytokines. A few studies have shown that bevacizumab exerts immunomodulatory effects when administered in combination with other anti-cancer drugs to patients affected by metastatic melanoma.^6^ In light of the close relationship between inflammation and angiogenesis, considerable interest has been aroused in the role of inflammation indexes to predict the efficacy of bevacizumab.

Systemic inflammation related indexes, such as Glasgow prognostic score (GPS), lymphocyte-to-monocyte ratio (LMR), neutrophil-to-lymphocyte ratio (NLR), and platelet-to-lymphocyte ratio (PLR) have been proved to be significantly associated with poor survival in several types of cancer, including colorectal cancer 7-10. We previously shown that NLR is an effective, and more accurate than platelet-to-lymphocyte ratio, to evaluate the survival in mCRC patients 11. However, there is few data explored relationship between inflammation-related factors and efficacy of bevacizumab 12. The current retrospective study was intending to evaluate the predictive value of systemic inflammation related indexes in mCRC patients receiving bevacizumab plus standard chemotherapy. If validated, these parameters could represent a reproducible, inexpensive and easy method to select candidates for treatment with bevacizumab.

## Methods

### Patients

Between January 2004 and September 2014, 1097 histologically confirmed and measurable stage IV CRC patients at Sun Yet-sen University Cancer Center were retrospectively reviewed. The main eligible criterion was that standard chemotherapy with or without bevacizumab should be administrated in the first-line therapy. Other criteria for eligibility were (1) ECOG performance scores (PS) of 0, 1, or 2, (2) adequate hepatic, renal and bone marrow function,(3) no inflammation related complications, (4) there are detailed pretreatment data on whole blood cell count, albumin, and c-reaction protein, (5) a life expectancy of > 3 months. Written informed consent was required before treatment.

Exclusion criteria included (1) anti-epidermal growth factor receptor (anti-EGFR) monoclonal antibodies were included in the first-line therapy, (2) metastatic lesions can be completely removed by local treatments, (3) patients in inflammatory conditions or treated with glucocorticoids or nonsteroidal anti-inflammatory drug, (4) no data on the time of progression, and (5) no clinical data available obtained.

This retrospective study was approved by the Institutional Review Board of the Sun Yat-sen cancer center. All inpatients were informed that their medical records may be reviewed for scientific research purposes and their individual confidentiality would be protected in accordance with the ethical standards of the Declaration of Helsinki.

### Data collection and category

Detailed baseline patients' characteristic data on age, sex, PS, initial stage, K-Ras status was collected based on medical records. Information on neutrophil counts, lymphocyte counts, monocyte counts, albumin and C-reaction protein (CRP) from blood tests carried out at baseline were also collected. The baseline CEA, CA199 and LDH were also collected. LMR was obtained by dividing the absolute lymphocyte count by the absolute monocyte count, NLR was calculated as the ratio of absolute neutrophil count to absolute lymphocyte count, and PLR was calculated as the ratio of absolute platelet count to absolute neutrophil count. According to previous reports, the cut-off of LMR, NLR and PLR was set as 3, 3.19and 169, respectively 12. The GPS is defined as follows: the presence of both elevated CRP (> 10 mg/L) and hypoalbuminemia (< 35 g/L) is given a score of 2 and the presence of only 1 parameter or neither of CRP and albumin abnormalities is given a score of 1 or 0, respectively.

### Statistical Analyses

Descriptive statistics were reported as proportion and medians. Tumor responses were assessed by RECIST 1.1 criteria every 6-8 weeks. Progression-free survival (PFS) was defined as the time from the initial of bevacizumab plus chemotherapy in the first-line treatment to disease progression or death. Overall survival (OS) was defined as the time from the initial of bevacizumab plus chemotherapy in the first-line treatment to death from any cause or loss of follow-up. Fisher's exact test and χ^2^ analysis were used, as appropriate, for categorical data. PFS or OS was calculated using the Kaplan-Meier method and data were compared using the log-rank test. All factors possibly influencing PFS or OS were evaluated using univariate and, subsequently, multivariate analyses. Values of 2-tailed P less than 0.05 were considered statistically significant. SPSS version 18.0 was used for statistical analysis.

## Results

### Patient characteristics

Between January 2004 and September 2014, 81 patients were treated with chemotherapy in combination with bevacizumab, and 213 with chemotherapy alone, which were included in the final analyses. All major clinical characteristics of two groups of patients were comparable. These clinical characteristics included age at diagnosis, sex, primary tumor, pathological grade, stage at diagnosis, K-Ras status, and proportions receiving second-line or third-line treatment (Table 1).

### Correlations between hematologic markers of systemic inflammation and the survival in chemotherapy alone group

**Figure 1:**
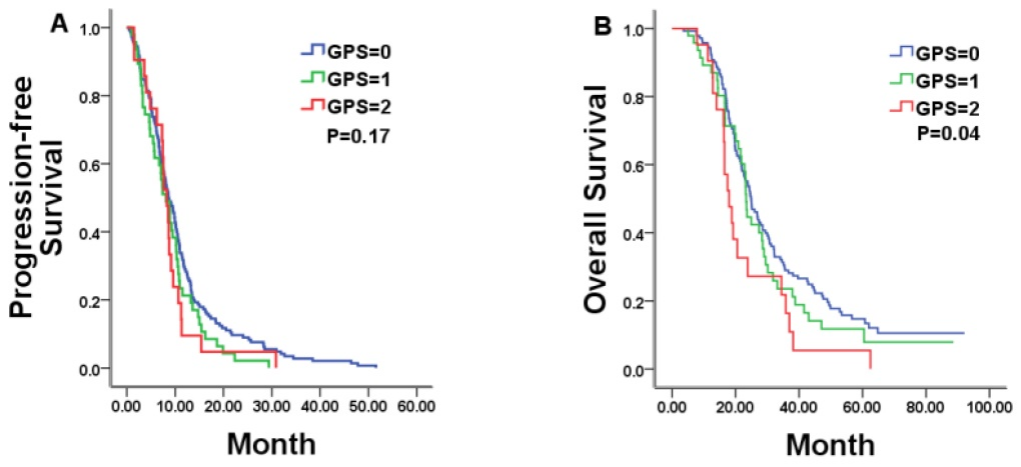
Kaplan-Meier estimates of median progression-free survival (PFS) and overall survival (OS) in mCRC patients with a GPS of 0, 1, and 2 treated by chemotherapy alone.

In all, 213 patients were available for analysis in the chemotherapy alone group with 8.57months of PFS and 23.80months of OS, respectively. In this group, pre-treatment GPS category was statistically related to OS, but not to PFS. Median PFS was 8.67, 8.10, and 8.27 months for patients with a GPS of 0, 1, and 2, respectively (P = 0.17; Figure 1A and Table 2). Patients with a GPS of 0, 1, and 2 achieved a median OS of 24.87, 23.30, and 17.93 months, respectively (P = 0.04; Figure 1B and Table 2). Among the other tested markers of systemic inflammation, median PFS was 8.77 and 7.43 months, respectively, for low- and high-PLR patients (P = 0.03). However, there was no significant difference in OS between the two group patients (median OS in low-PLR vs. high-PLR patients: 24.97 vs.21.53 months, respectively, P = 0.28).

### Correlations between hematologic markers of systemic inflammation and the survival in bevacizumab group

**Figure 2:**
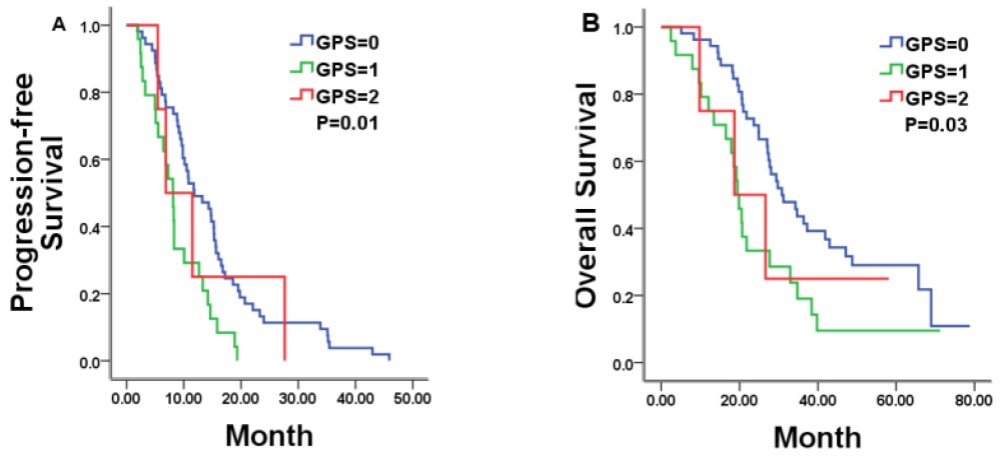
Kaplan-Meier estimates of median PFS and OS in mCRC patients with a GPS of 0, 1, and 2 treated by bevacizumab (Bev) plus chemotherapy.

In total, 81 patients were available for analysis in the bevacizumab plus chemotherapy group with 10.13 months of PFS and 27.67 months of OS, respectively. In this group, pre-treatment GPS category was statistically related to both PFS and OS. Patients with a GPS of 0, 1, and 2 achieved a median PFS of 11.83, 8.10, and 6.87 months, respectively (P = 0.01; Figure 2A and Table 2). Median OS was 30.80, 19.47, and 18.67 months for patients with a GPS of 0, 1, and 2, respectively (P = 0.03; Figure 2 Band Table 2). Other tested markers of systemic inflammation (LMR, NLR and PLR) failed to show a significant correlation with the PFS or OS (Table 2).

### Correlations between hematologic markers of systemic inflammation and the survival in the whole group

**Figure 3:**
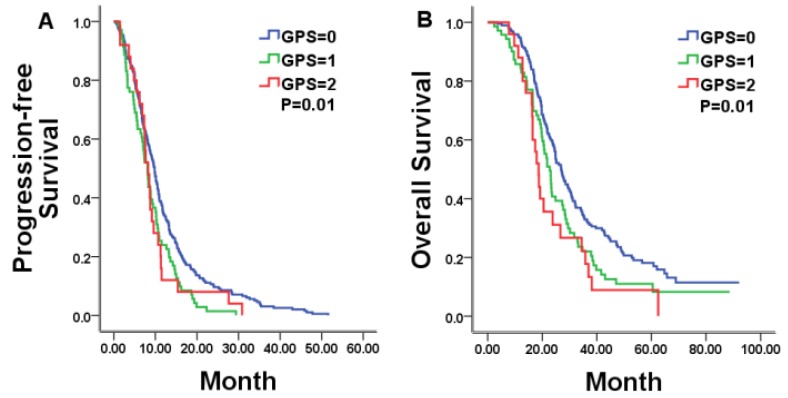
Kaplan-Meier estimates of median PFS and OS in whole mCRC patients with a GPS of 0, 1, and 2.

**Figure 4:**
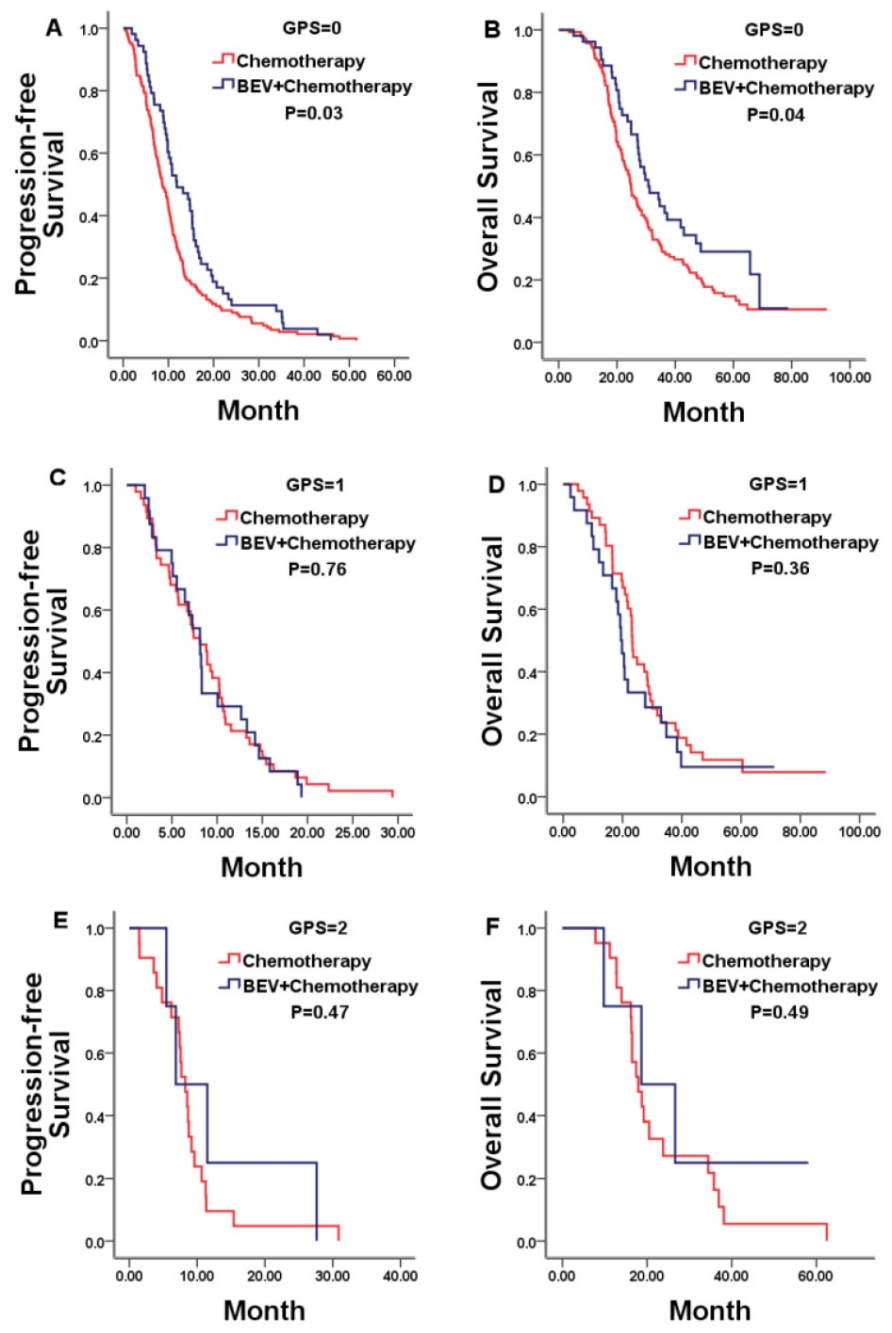
Kaplan-Meier estimates of median PFS and OS in mCRC patients with a GPS of 0, 1, and 2 treated by bevacizumab (Bev) plus chemotherapy or chemotherapy alone. No statistically significant differences were noticed for PFS or OS in patients with a GPS of either 1 or 2.

We firstly explored the prognostic value of markers of systemic inflammation in the whole group. In according with previous reports in unresectable mCRC, the GPS was a reliable negative prognostic factor for OS, but high NLR/PLR and low LMR was not significantly related to shorter OS. Median OS was 26.97, 22.77, and 18.67 months for patients with a GPS of 0, 1, and 2, respectively (P = 0.01; Figure 3B).Then, we compared results from the bevacizumab and chemotherapy alone groups according to pre-treatment markers of systemic inflammation. Only in patients with a GPS of 0, both PFS and OS was in favor of patients treated with bevacizumab plus chemotherapy compared with who treated with chemotherapy alone (PFS 11.83 vs. 8.67 months, p=0.03; OS 30.80 vs. 24.87 months, p=0.04; Figure 4A, 4B). No statistically significant differences were noticed for overall survival in patients either with a GPS of 1, 2 or with different NLR/PLR/LMR levels. This relationship between GPS and treatment effect suggested that good GPS was a predictor of improved survival associated with the effect of bevacizumab.

## Discussion

Hematologic markers of the systemic inflammatory response have been proved to be significantly associated with the cancer-specific survival in many types of cancer 13. In a specific setting of a certain cancer, however, the result may be biased by retrospective nature of the study, heterogeneity of patient characteristics, and inflammation-related comorbidities 14. In unresectable mCRC, the low GPS seems to be a reliable favorable prognostic factor in most of studies, no matter what regimens are administrated for these patients 15, 16. The present study confirmed that GPS is a reliable prognostic marker regarding OS in patients with unresectable mCRC. Median OS was 26.97, 22.77, and 18.67 months for patients with a GPS of 0, 1, and 2, respectively (P = 0.01). Other inflammation markers, such as LMR, NLR and PLR, were not proven as the prognostic factors for the patients with unresectable mCRC in the present study. In previous studies, patients with high NLR and low LMR seems to have shorter PFS and OS, but the data wasn't insufficient 12. PLR was also not supported as an independent predictor of either OS or PFS in mCRC 17, 18.

What's important is that results from the present study support the use of GPS as a predictor of clinical benefit in patients with mCRC receiving first-line bevacizumab plus chemotherapy. GPS of 0 was able to identify a subset of patients most likely to benefit from bevacizumab plus chemotherapy in PFS and OS. Maillet and colleagues analyzed the prognostic and predictive value of GPS in mCRC patients receiving bevacizumab 19. Their data suggested that higher GPS is a poorer prognostic marker regarding OS in patients treated with bevacizumab plus chemotherapy. They postulated that the use of the GPS could help in better identifying mCRC patients who could derive benefit from intensive treatments. Because there was no comparative group with chemotherapy alone, they couldn't draw a convincing conclusion 19. To our knowledge, this is the first study to describe the use of GPS to provide predictive information regarding bevacizumab in the first-line palliative treatment setting for patients with unresectable mCRC.

The reasons for GPS to predict benefit from bevacizumab remain unknown. Bevacizumab, as the monoclonal antibody targeting VEGF, plays the anti-cancer role by inhibiting tumor angiogenesis. However, studies reveal that VEGF facilitates tumor immune evasion and intolerance through different mechanisms 20. Therefore, bevacizumab may also plays the anti-cancer role by overcoming immune evasion and intolerance through several mechanisms. These mechanisms include increasing CD8+ T cell infiltration and reducing T regulatory cells and myeloid-derived suppressor cells (MDSCs) infiltration in cancer nest, and reducing suppressive cytokines 21,22. Data from phase IIIITACa (Italian Trialin Advanced Colorectal Cancer) trial suggested that patients with good systemic inflammation status (NLR < 3) in the bevacizumab plus chemotherapy arm had a higher PFS than those treated with chemotherapy alone, but bad systemic inflammation status(NLR ≥ 3) was associated with a poor prognosis 23,24. These results imply that good systemic inflammation status may be correlated with a favorable immunological effect of bevacizumab.

There were also several limitations associated with the present study. First, due to the study's retrospective nature, selection bias cannot be excluded in the study. Second, the sample of this study is relatively small. Third, we couldn't validate our findings because of lacking an independent cohort, which made our finding less convincing. However, a Japanese study also found that patients with GPS 0 has better survival in all bevacizumab treatment 25. This may confirm our finding to some extent. Last, we used previously reported cut-off points to analysis data, however, the reasonable cut-off values for markers of systemic inflammation remain unknown.

## Conclusion

Our data showed that GPS is reliable prognostic index in mCRC patients. Furthermore, GPS of 0 was able to identify a subset of patients most likely to benefit from bevacizumab plus chemotherapy. Prospective studies need to confirm these results.

## Figures and Tables

**Table 1:** Patient characteristics

Variables	Bevacizumab group	Chemotherapy group	P value
**Age, years**			0.16
Median (range)	48 (24-77)	55 (23-81)	
≤ 65	68 (84.0%)	170 (79.8%)	
> 65	13 (16.0%)	43 (20.2%)	
**Sex**			0.42
Female	35 (43.2%)	73 (34.3%)	
Male	46 (56.8%)	140 (65.7%)	
**Primary tumor**			0.25
Left-sided	53 (65.4%)	154 (72.3%)	
Right-sided	28 (34.6%)	59 (27.7%)	
**Pathological grade**			0.17
Well differentiation	4 (4.9%)	11 (5.2%)	
Mild differentiation	45 (55.6%)	123 (57.7%)	
Poor differentiation	13 (16.0%)	44 (20.7%)	
Mucinous adenocarcinoma	19 (23.5%)	31 (14.6%)	
**Stage at the first diagnosis**			0.41
I/II/III	26 (%)	58 (27.2%)	
IV	55 (61.8%)	155 (72.8%)	
**K-Ras status**			0.001
Wild type	23 (28.4%)	47 (22.1%)	
Mutated type	21 (25.9%)	23 (10.8%)	
Unknown	37 (45.7%)	143 (67.1%)	
**CEA**			0.03
≤ 5ng/mL	16 (19.8%)	69 (32.4%)	
> 5 ng/mL	65 (80.2%)	143 (67.1%)	
**CA199**			0.33
≤ 37U/mL	35 (43.2%)	93 (43.7%)	
> 37U/mL	42 (51.9%)	120 (56.3%)	
**LDH**			0.92
≤ 245U/L	59 (72.8%)	154 (72.3%)	
> 245U/L	22 (27.2%)	59 (27.7%)	
**LMR**			0.86
≤ 3	31 (38.2%)	85 (39.9%)	
> 3	50 (61.7%)	128 (60.1%)	
**NLR**			0.28
≤ 3.19	60 (74.1%)	143 (67.1%)	
> 3.19	21 (25.9%)	70 (32.9%)	
**PLR**			0.63
≤ 169	50 (61.7%)	137 (64.3%)	
> 169	31 (38.2%)	76 (35.7%)	
**GPS**			0.21
0	53 (65.4%)	145 (68.1%)	
1	24 (29.6%)	47 (22.1%)	
2	4 (4.9%)	21 (9.8%)	
**2nd line treatment**	63 (77.8%)	170 (79.8%)	0.70
**3rd or later line treatment**	26 (32.1%)	81 (38.0%)	0.35

Abbreviations: GPS = Glasgow Prognostic Score; LMR = lymphocyte-to-monocyte ratio; NLR = neutrophil-to-lymphocyte ratio; PLR = platelet-to-lymphocyte ratio.

**Table 2:** Univariate overall and progression-free survival analysis in mCRC patients with different pre-treatment inflammatory response

	Variables	Chemo group HR (95%CI)	Bev+Chemo group HR (95%CI)	Whole group HR (95%CI)
**PFS**	LMR (≤ 3 vs. > 3)	1.17(0.89-1.54)	1.23(0.77-1.94)	1.17(0.93-1.48)
	NLR (≤ 3.19 vs. >3.19)	0.93(0.70-1.24)	0.91(0.55-1.51)	0.92(0.71-1.18)
	PLR (≤169 vs. >169)	***0.73(0.55-0.97)***	0.83(0.53-1.31)	***0.77(0.60-0.98)***
	GPS (0 vs. 1/2)	0.76(0.57-1.02)	***0.50(0.31-0.80)***	***0.67(0.53-0.86)***
**OS**	LMR (≤3 vs. >3)	1.31(0.97-1.77)	1.19(0.71-2.01)	1.27(0.98-1.65)
	NLR (≤3.19 vs. > 3.19)	0.80(0.58-1.08)	0.85(0.47-1.53)	0.80(0.61-1.05)
	PLR (≤169 vs. > 169)	0.84(0.62-1.15)	0.73(0.42-1.22)	0.82(0.63-1.07)
	GPS (0 vs. 1/2)	0.75(0.55-1.02)	***0.50(0.30-0.84)***	***0.67(0.51-0.87)***

Abbreviations: Bev = bevacizumab; Chemo = chemotherapy; CI = confidence interval; GPS = Glasgow Prognostic Score; HR = Hazards Ratio; LMR = lymphocyte-to-monocyte ratio; NLR = neutrophil-to-lymphocyte ratio; PLR = platelet-to-lymphocyte ratio; PFS = progression-free survival; OS = overall survival.

## Abbreviations

Anti-EGFR: anti-epidermal growth factor receptor
CI: confidence interval
CRP: C-reaction protein
GPS: Glasgow prognostic score
LMR: lymphocyte-to-monocyte ratio
mCRC: metastatic colorectal cancer
MDSCs: myeloid-derived suppressor cells
NLR: neutrophil-to-lymphocyte ratio
OS: overall survival
PFS: progression-free survival
PLR: platelet-to-lymphocyte ratio
PS: performance scores
